# Fine-Tuning the Physicochemical Properties of Poly(lactic Acid) Nanoparticles for the Controlled Release of the BET Inhibitor JQ1: Influence of PVA Concentration

**DOI:** 10.3390/polym17010123

**Published:** 2025-01-06

**Authors:** Nedjla Kedjar, Eleonora Iannuzzi, Martin Kreuzer, Carlos Alonso-Moreno, Carmen Moya-Lopez

**Affiliations:** 1 Laboratory of Applied Chemistry (LAC), Faculty of Sciences Technology, University of Ain Temouchent Belhadj Bouchaib, Ain Temouchent 46000, Algeria; njkedj@gmail.com; 2 Facultad de Farmacia-Centro de Innovación en Química Avanzada (ORFEO-CINQA), Unidad nanoDrug, Departamento de Química Inorgánica, Orgánica y Bioquímica, Universidad de Castilla-La Mancha, 02071 Albacete, Albacete, Spain; eleonora.iannuzzi@uclm.es; 3 ALBA Synchrotron, Carrer de la Llum 2-26, 08290 Cerdanyola del Vallès, Barcelona, Spain

**Keywords:** PLA, polymeric nanoparticles, double emulsification, JQ1

## Abstract

The compounds targeting the bromo and extra terminal domain proteins (BET), such as the JQ1, present potent anti-cancer activity in preclinical models, however, the application of JQ1 at the clinical level is limited by its short half-life, rapid clearance, and non-selective inhibition of BET family proteins, leading to off-target effects and resistance. To address these challenges, the optimization of JQ1 delivery has been accomplished through polylactide (PLA) nanoparticles. PLA derivatives with varying molecular weights were synthesized via ring-opening polymerization using a zinc-based initiator and characterized using thermogravimetric analysis, differential scanning calorimetry, and infrared spectroscopy. PLA nanoparticles (NPs) were subsequently formulated, and the effects of key parameters—including PLA molecular weight, organic phase concentration, and surfactant concentration—on particle size, polydispersity index (PDI), and encapsulation efficiency were systematically investigated. PLA molecular weight and organic phase concentration mainly influenced the NPs size whilst the thermodynamic state of the NPs was unaffected by these two parameters. The surfactant concentration is correlated to the encapsulation efficacy of JQ1 as well as the release profile, suggesting the potential tool that the variation of these parameters represent for customizing the release of JQ1 according to specific needs.

## 1. Introduction

Triple-negative breast cancer (TNBC) is an aggressive subtype that accounts for ca. 15% of all breast tumors and is associated with poor prognosis and a high rate of recurrence. TNBC is characterized by the absence of estrogen receptors, progesterone receptors, and HER2 receptors, which limits the efficacy of targeted therapeutic agents [1]. To address these challenges, novel strategies are being explored, such as the development of rapid breast cancer screening using Raman-based analysis [2,3]. Additionally, the development of innovative drugs targeting the epigenome of TNBC cells is also under investigation. Among these, compounds targeting the bromo and extra terminal domain proteins (BET), such as JQ1, have emerged as promising candidates and are currently under clinical development [4,5]. However, its widespread use is restricted by various factors. These include the development of acquired resistance due to compensatory pathways, limited long-term tolerability, and off-target effects on non-tumor tissues expressing the target protein. Furthermore, the highly lipophilia related to its chemical structure causes poor solubility which hinders cell permeability and therefore could require the use of higher doses for the treatment of patients. Such events might result in significant non-tumor toxicity and adverse effects on patients [6]. In this context, nanomedicine offers a promising approach, potentially overcoming several of such existing drawbacks. By encapsulating JQ1 within nanoparticles (NPs), its delivery and pharmacokinetic profile can be enhanced, allowing for targeted delivery to tumor sites. This approach has the potential to overcome chemotherapy resistance, minimize systemic toxicity, and improve therapeutic outcomes. However, the development of drug delivery systems (DDS) remains in its early stages, requiring further validation and optimization. Key challenges include increased complexity, high costs associated with carrier materials and pharmaceutical excipients, and the need for rigorous assessment of safety and efficacy.

Designing innovative nanomedicines for rapid clinical translation remains a significant challenge. To date, only a limited number of nanomedicines have received clinical approval for cancer treatment. Among the various nanosystems, polymeric NPs have emerged as a leading drug delivery platform due to their exceptional biocompatibility, high bioavailability, and ability to carry multiple therapeutic payloads [7]. One of the most promising materials for developing polymeric-based NPs is the Food and Drug Administration (FDA)-approved poly(lactic acid) (PLA) [8]. Its advantageous physicochemical properties along with its low price made it an environmentally-friendly alternative to traditional petroleum-derived polyolefins. Furthermore, PLA is highly biocompatible, as its hydrolysis in physiological conditions generates lactic acid, an endogenous molecule that is naturally metabolized into water and carbon dioxide. These features position PLA as an ideal candidate for the development of advanced nanocarrier systems for cancer therapeutics [9].

The physicochemical properties of PLA depend on different parameters such as stereoregularity architecture or molecular weight, which can be tuned by controlling the polymerization process, offering the possibility to tailor the properties of the final material for specific applications [10]. In particular, the FDA approved the utilization of PLA for biomedical purposes back in 1960, reaching the clinical level for several applications such as sutures, dermal fillers, tissue engineering, or DDSs [11,12,13]. The design of DDSs based on PLA derivatives allows tuning the encapsulation and loading efficiency, the release rate of the drug, etc. by modifying the synthesis and post-synthesis processing of PLA [14]. Nowadays, more than 15 PLA-based microparticles have already reached the market to control drug delivery [11] and in particular, PLA microparticles have been applied for the treatment of several pathologies such as cancer [15,16] or insulin-dependent diabetes [17]. However, the absence of a clinical application for PLA nanoparticles despite the research abundance on the drug load/release might be due to the lack of know-how transfer between the fundamental research on the DDS design and clinical trials, which might be due to a lack of understanding over the formulation process. The lack of robustness and reproducibility during the nanoformulation of PLA at laboratory scale hampers the successful transfer from research to clinical application [18,19].

Numerous factors contribute to forming the properties of micro- and nanoparticles [20] and several studies showed contradictory results for the same parameter-property relationship [21,22]. Particularly, the nanoparticle size was found to decrease [23] or increase [24] with increasing the PLA molecular weight. Therefore, novel formulation approaches focussing on a deeper understanding of the nanoparticle formation mechanism as well as its final thermodynamical state depending on the processing conditions are necessary to control the drug delivery process such as the initial burst release [19,20].

The conversion of JQ1 into nanomedicines has been scarcely explored. Previous studies have demonstrated that encapsulating JQ1 in polymeric NPs can reduce the expression of c-Myc mRNA in tumor tissues more effectively than free JQ1 [25]. Moreover, liposomal NPs [26] were also formulated to encapsulate JQ1, as well as poly(ethylene glycol)-poly(aspartic acid)-NPs [27] and Zein NPs [28]. Despite these advancements, the impact of formulation parameters on the properties of NPs is often overlooked. Inadequate control of these parameters can lead to significant variability in the properties of the final formulation [20], potentially compromising its efficacy and reproducibility. Therefore, a deeper understanding of how formulation and process parameters influence NPs properties is essential for optimizing JQ1-based nanomedicines.

In this study, a series of PLA derivatives with varying molecular weights were synthesized via ring-opening polymerization (ROP) using an organometallic zinc-based initiator, allowing for tuning the properties of the polymer. The physicochemical characteristics of the PLA derivatives were analyzed using thermogravimetric analysis (TGA), differential scanning calorimetry (DSC), and infrared spectroscopy (FTIR). Subsequently, PLA-based NPs were formulated, and the influence of three key parameters—PLLA molecular weight, organic phase concentration, and excipient concentration—on the final NPs properties was systematically investigated. While the complexity of process development requires consideration of numerous factors, this study focused on these parameters and their effects on particle size, polydispersity index (PDI), and thermodynamic state. Additionally, the encapsulation efficiency, drug loading, and release profile were evaluated as critical variables.

This targeted approach provides insights into optimizing formulation parameters for the development of advanced polymeric nanocarrier systems.

## 2. Materials and Methods

### 2.1. General

Solvents and reagents were acquired from Sigma-Aldrich (St. Louis, MO, USA) apart from L-lactide (L-LA) monomers that were purchased from Rex Scientific (China). The lactide monomers were purified three times by sublimation and stored in a glovebox at 4 °C. Toluene was pre-dried over sodium wire as well as distilled under nitrogen conditions from sodium and subsequently stored over molecular sieves (3 Å) in a glovebox. Phosphate buffered saline (PBS, pH 7.4), Spectra/Por 2 Dialysis Membrane MWCO: 12–14 kDa, and Poly(vinyl) alcohol (PVA, mol wt 31,000–50,000, 98–99% hydrolyzed) were purchased from Sigma-Aldrich Spain. JQ1 was purchased from MedChemExpress (Monmouth Junction, NJ 08852, USA).

### 2.2. Synthesis

All the synthetic manipulations were performed under nitrogen, using standard Schlenk techniques.

Synthesis of the initiator. The synthesis of the initiator [Zn(Et)(*κ*^3^-bpzteH)] (bpzteH = 2,2-bis(3,5-dimethylpyrazol-1-yl)-1-para-tolylethoxide] followed the previously published procedure [29].

General procedure for solution polymerization of LA. ROP was performed on a Schlenk line in a dried Schlenk flask equipped with a magnetic stirrer [29]. The Schlenk tubes were loaded in the glovebox with the required amount of LA and initiator, separately, and dissolved in the appropriate amount of solvent. Methanol was used to terminate the reaction and precipitate the synthesized polymer. The obtained polymers were collected by filtration, dried at room temperature, exposed to vacuum over 24 h in the Schlenk line, and stored upon characterization. The PLA-derivatives named PLLAXX, were XX corresponds to the approximate molecular weight in kDa. NMR Spectra were recorded at room temperature on a Varian Inova FT-400 spectrometer and referenced to the standard in the deuterated solvent with the relaxation time fixed to 4 s. The PLA derivatives were dissolved in CDCl_3_ for NMR characterization.

### 2.3. Nanoparticles Formulation

Empty PLA nanoparticles (PLA-NPs) were formulated using the four PLA derivatives, PLA32, PLA56, PLA71 and PLA98 by a double emulsion methodology [6,30]. Briefly, PLA (10, 20 or 30 mg) was dissolved in 4 mL of CH_2_CL_2_ and 2 mL of mQ water was added; then, the mixture was shaken in a vortex and subsequently sonicated in an ice bath for 1 min to avoid emulsion overheating. The pre-emulsion generated was added to 10 mL of polyvinyl alcohol (PVA) of different concentrations, shaken in a Vortex and sonicated for 5 min. The emulsion was allowed to evaporate the organic solvent at room temperature for 45 min in a fume hood. Subsequently, the samples were centrifuged at 4 °C and 15 k rpm for 20 min. NPs were collected and dissolved in mQ water.

The formulation of nanoparticles encapsulating JQ1 followed the same procedure as empty nanoparticles and 1 mg of JQ1 was added to the organic phase.

### 2.4. Polymer and Polymeric NPs Characterization

#### 2.4.1. Nanoparticle Size and Morphology of NPs

Nanoparticle size, polydispersity index and Z-potential were analyzed using the dispersion light scattering technique (DLS) on a Zetasizer Nano ZS instrument (Malvern Instruments, Malvern, UK). Data were analyzed using the multimodal number distribution software supplied with the instrument.

#### 2.4.2. Stability Studies of Formulations

For the stability evaluation of NPs, they were stored as an aqueous suspension at 4 °C and heated up to room temperature before the stability analysis. The hydrodynamic radius (R_H_) and polydispersity index (PdI) were periodically measured using dynamic light scattering (DLS) over up to 4 weeks.

#### 2.4.3. Encapsulation and Loading Efficiencies

NPs were dialyzed in a phosphate-buffered saline medium for 20 min to remove the unencapsulated drug with a dialysis membrane (MWCO: 12–14 kDa). The loading efficiency (LE%) and encapsulation efficiency (EE%) of the polymeric NPs were subsequently determined using the destruction method [30], applying the following equations:

LE% = (weight of encapsulate JQ1 (mg))/(weight of total (JQ1 encapsulated + scaffold weight) (mg)) × 100

EE% = (weight of encapsulated JQ1 (mg))/(initial JQ1 weight (mg)) × 100

#### 2.4.4. In Vitro Drug Release

300 µL of JQ1-loaded NPs were sealed in a dialysis membrane (MWCO: 12–14 kDa) and suspended in 15 mL PBS (pH 7.5) at 37 °C with stirring. 1 mL of release medium was taken at various intervals. The concentration of JQ1 released was determined by HPLC (Agilent 1200 Infility II, DAD detector, Agilent, Santa Clara, CA 95051, USA).

#### 2.4.5. Molecular Weight Determination

Molecular weight (Mn, Mw) as well as polydispersity index (PDI) of the PLA-derivatives, were determined from GEL Permeation Chromatography (GPC) using chloroform as eluent. The molecular weight characterization was performed with a Shimadwu Prominence-I LC-2030 equipped with a Shodez GPC KF-805L column (Shodex, Tokyo, Japan) and a Shimazu RIF-20A detector (Shimazu, Kyoto, Japan). Analytical grade CH_2_Cl_2_ was used as the mobile phase at 40 °C, with a flow rate of 1 mL/min. GPC samples were prepared by dissolving 5 mg of the corresponding polymer in 1.5 mL of solvent and left overnight under constant agitation. The samples were filtered over a 0.2 μm polytetrafluoroethylene syringe filter before its injection. Polystyrene standards were used a reference to determine the relative molecular weight of the samples.

#### 2.4.6. Thermal Stability Analysis

TGA measurements. The mass loss of the PLA-derivatives and PVA was analysed in the TGA Q50 from TA instruments (New Castle, DE, USA) with a range temperature from 25 °C to 1150 °C. Briefly, 1–4 mg of the corresponding polymer was loaded in the TGA pans and heated from 30 °C to 700 °C at a constant heating rate of 10 °C/min.

DSC measurements. DSC analysis was performed using a Q20 DSC (Ta Instruments, New Castle, DE, USA). The calibration was performed using indium as a standard (T_m_ = 156.61 °C). DSC samples were prepared by sealing around 5 mg of the samples in Tzero Hermetic aluminium pans (TA Instruments, New Castle, DE, USA). The thermal protocol performed for bulk polymers consisted of a first heating ramp at 10 °C/min from 25 °C to 200 °C, followed by a cooling ramp at 10 °C/min to 25 °C. The thermal transitions (T_g_, T_c_ and T_m_) and their corresponding enthalpies (ΔH_c_, ΔH_m_) were calculated from the obtained thermograms.

Time-resolved FTIR. The transmission-type Fourier-transform infrared spectra (FTIR) were measured at BL01 MIRAS at Alba Synchrotron, equipped with a Hyperion 3000 microscope coupled to a Vertex 70 spectrometer (BRUKER, Germany) [31]. Temperature ramps were done using a Linkam stage at a heating rate of 10 °C/min. Infrared spectra were recorded continuously with 32 accumulated scans per spectrum using a nitrogen cooled MCT detector. The spectra were baseline corrected and unit-vector normalized in the regions of interest with the Quasar software package Version 1.10.1 [32].

## 3. Results

### 3.1. Synthesis and Characterization of PLA Derivatives

**Figure 1:**
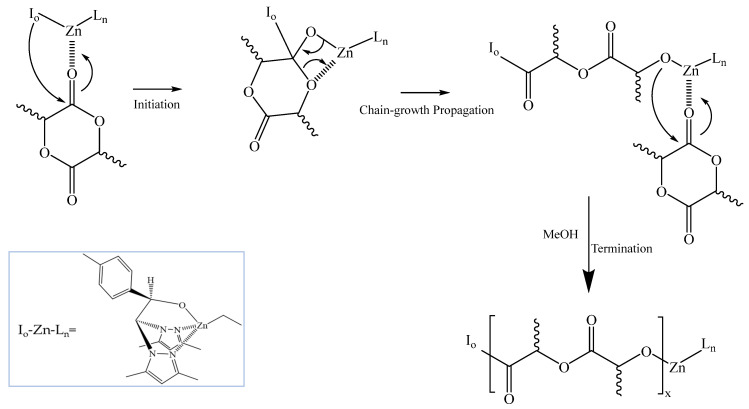
Proposed mechanism for the ROP of lactide using the [Zn(Et)(*κ*^3^-bpzteH)] (bpzteH = 2,2-bis(3,5-dimethylpyrazol-1-yl)-1-para-tolylethoxide].

The synthesis of the PLA derivatives was accomplished by the ROP of the L-lactide monomers using a heteroscorpionate initiator as previously described [29] (Figure 1). The ROP reaction conditions were optimized based on prior experience. The successful polymerization of PLA was confirmed via ^1^H NMR spectroscopy (Figure S1 in the Supporting Information). Molecular weight variations in the synthesized polymers were controlled by adjusting the [LA]/catalyst ratio. Monomer conversions were higher than 80% as proved by the calculated PLA/LA ratios derived from the ^1^H NMR (Figure S2 in the Supporting Information). Additionally, molecular weight values determined by GPC were in good agreement with the calculated values (Table 1), confirming the accuracy of the polymerization process (Figures S3–S6 in the Supporting Information).

**Figure 2:**
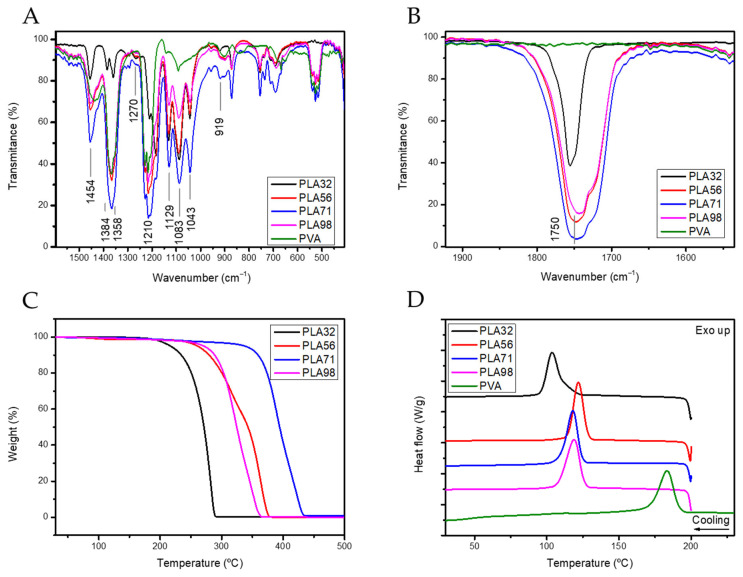
FTIR spectra of the PLA derivatives and PVA in the (**A**) 1600–400 cm^−1^ and (**B**) 2000–1650 cm^−1^ regions. (**C**) TGA thermogram of the synthesized PLA derivatives at a heating rate of 10 °C/min. (**D**) Heating thermograms of the synthesized PLA-derivatives and PVA at a heating rate of 10 °C/min.

FTIR analysis was conducted to confirm the chemical composition of the synthesized PLA derivatives and the excipients used in the formulations, such as PVA, that will be used eventually as a comparison for the temperature-resolved FTIR analysis. The key functional groups involved in the thermal transitions were identified (Figure 2A,B) and summarized in Table S1 of the Supporting Information. The most characteristic vibrational bands of PLA include the C-H asymmetric vibration at 2993 cm^−1^, the carbonyl stretching vibration at 1750 cm^−1^ which was absent in the PVA spectrum, the asymmetric vibration of the C-O-C bond related to the amorphous phase at 1270 cm^−1^ as well as the vibration associated to the C-H + C-O-C interaction at 1210 cm^−1^ which is associated with the interaction occurring in PLA previous to the crystallization. Additionally, understanding the thermal degradation of the PLA derivatives is essential for evaluating how thermal treatments might impact their stability. Thermal degradation of the PLA derivatives was analysed by following the loss mass during a 10 °C/min heating ramp from room temperature to 700 °C by TGA (Figure 2C and Figure S7 of the Supporting Information). The PLA-derivatives started to degrade at a temperature higher than 200 °C (Table 2), presenting a higher starting degradation temperature as the molecular weight increased, except for the PLA98, which presented a degradation temperature like PLA56. The decrease in the degradation temperature of the PLA98 might be ascribed to a hampered purification step due to the increase of the molecular weight that increases the entanglement degree and therefore might impede the complete purification of the polymer. Therefore, the presence of non-polymerized monomers and catalyst within the sample might decrease the degradation temperature [33].

DSC experiments were conducted on bulk polymers to understand the effect of the PLA molecular weight on the thermal transitions (Figure 2D and Table 2). The glass transition temperature of PLA is around 70 °C independently of the Mw, although slightly higher T_g_ is observed as the Mw increases. The glass transition is ca. 10 °C higher than the T_g_ of commercial PLA [34] probably due to the absence of the additives that are usually included in the commercial PLA pellets and act as plasticizers.

Interestingly, the endothermal transition upon heating the PLA71 starting at 160 °C is a double melting peak, suggesting the presence of two crystal phases (α- and α’-crystal phases), whilst for lower Mw (PLA56 and PLA33) the percentage of PLA crystallized in the α’ crystal phase might be lower since the endothermal transition before the main melting peak is smaller. This event might be due to the higher mobility of the polymeric chains due to the lower molecular weight that enables the crystallization in the more perfect crystal phase, the α-phase [34,35], since the crystallization rate is significantly affected by PLA Mw [36]. However, the double endothermal transition exhibited in the high molecular weight PLA could be also ascribed to a slower transition of the α’- to the α-, since it is favoured in the low molecular weight samples [37]. Moreover, the glass transition temperature of PVA was 46.70 °C which is 40 °C lower than the T_g_ of PVA previously reported [38], but similar to the PVA T_g_ under humid conditions (41 °C of PVA of 15 kDa [39,40]).

### 3.2. Formulation of PLA-Derived NPs

**Figure 3:**
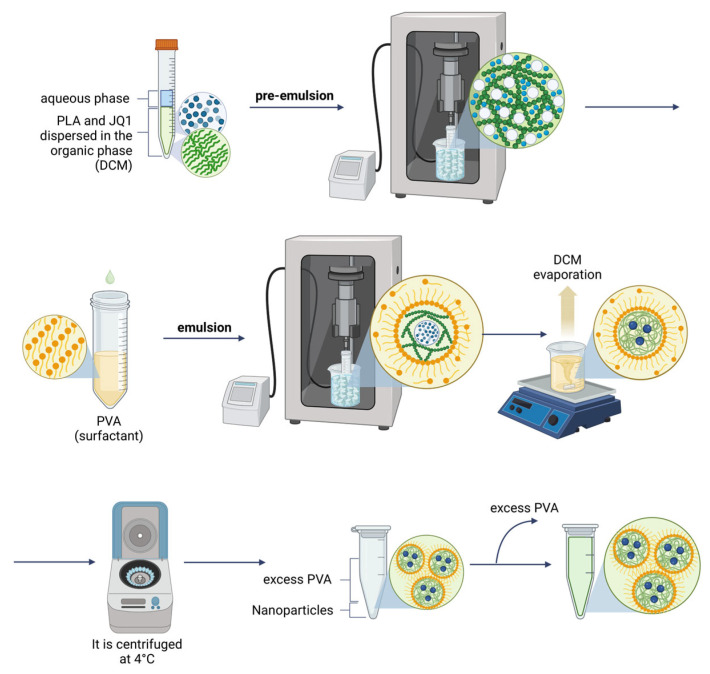
Schematic representation of the workflow for the formulation of PLA-based NPs by solvent diffusion method.

PLA-based NPs were prepared using a double emulsion technique (Figure 3), with PVA as a surfactant. In general, DLS analysis showed a R_H_ of approximately 150 nm, which is in the range of previously formulated nanoparticles [41], as well as monodisperse formulations and polydispersity index (PdI) values near 0.1, indicating high homogeneity. The Zeta-potential values were in the range of ca. −20 mV which determines the high stability of the formulations since as the zeta potential increases, the repulsive interactions between nanoparticles are stronger preventing aggregation [41]. The NPs exhibited excellent stability, attributed to their pronounced negative surface charge (Table 3).

**Figure 4:**
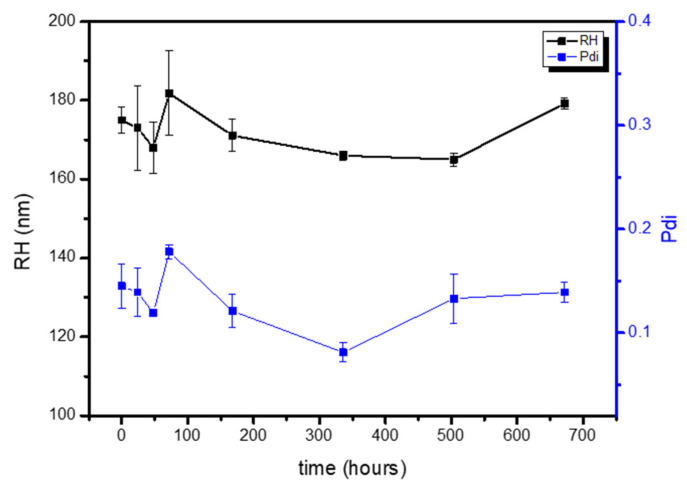
PLA-based NPs stability.

The physical stability of PLA-NPs was also studied for one month stored at 4 °C and the negligible increase in either particle size or PdI denoted high stability against aggregation (see Figure 4 as a representative example of the stability of the PLA-derived formulations).

The effect of different parameters, such as the PLA-molecular weight, the PVA concentration, and the organic phase concentration, on the NPs physicochemical properties was analysed.

#### 3.2.1. Effect of PLA Molecular Weight in NPs Formulation

The R_H_ of the PLA-NPs increased from ca. 150 nm to ca. 176 nm as the molecular weight of the PLA increased from 33 kDa to 98 kDa (Table 3). This behaviour contrasts with previous reports on similar molecular weight increases [23], but aligns with other reported analyses [24]. The Z-Potential values were in the range of ca. −20 mV independently of the M_w_ and the lowest PDI value was found for the lower M_w_ formulation. Moreover, the T_g_ of the PLA-NPs increased from 57 °C to 59 °C as the molecular weight increased (Table 4), remaining 10 °C lower than the T_g_ of raw PLA [42]. This reduction is likely ascribed to the plastification effect of the solvents used during formulation, which enhances chain mobility. In addition, PLA-NPs exhibited cold crystallization upon heating at ca. 80 °C (Table 4), unlike raw PLA, indicating that the PLA does not fully crystallize during the NPs formulation probably ascribed to the rapid formulation rate. The cold crystallization temperature (T_cc_) also increased as the molecular weight increased, reflecting the higher mobility required for crystallizing as the molecular weight increased. The melting temperature (T_m_) of the PLA-NPs was found to be around 172 °C, presenting a mono-distributed form (α or α’-phase) independently of the molecular weight and contrary to the double melting endotherm observed in the raw PLA at higher molecular weights.

**Figure 5:**
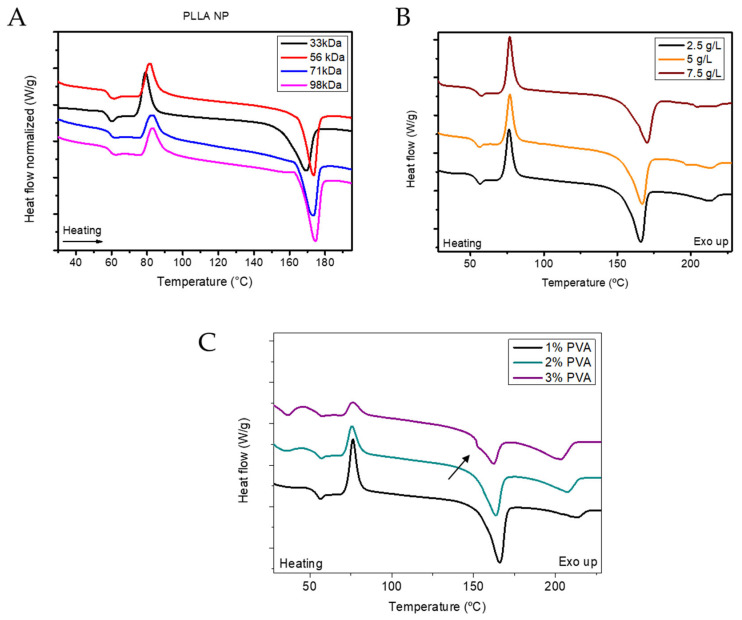
DSC thermogram upon heating at 10 °C/min of the PLA-NPs as a function of PLA Molecular weight (**A**), PLA concentration (**B**) and PVA concentration (**C**).

The variation in molecular weight had a significant effect on the size of the NPs. In addition, the formulation process unified the physical-chemical state of the NPs, as observed by DSC (Figure 5A), since T_g_, T_cc_ and T_m_ values were similar independently of the molecular weight, and significantly different to the values exhibited by raw PLA. Since the nanotechnology objective is the fabrication of NPs in the nano-range, the formulations with the smallest particle size were selected for further optimization.

#### 3.2.2. Effect of the Polymer Concentration

The effect of the polymer concentration on the size and size distribution of the NPs formulated from PLA33 at a PVA concentration of 1% was evaluated. The polymer concentration was varied from 2.5 g/L to 7.5 g/L and the R_H_ of the PLA-NPs increased from ca. 149 nm to ca. 168 nm (Table 5), similar to what was elsewhere described for PLGA-NPs formulated both by nanoprecipitation [41] or emulsion [42]. The PDI values did not significantly vary as a function of polymer concentration although the Z-potential decreased as the polymer concentration increased likely ascribed to the lower PVA/PLA ratio as the polymer concentration increased.

In addition, the thermodynamical state of the NPs after formulation was similar independently of the polymer concentration used, as observed by DSC analysis (Table 6 and Figure 5B). The T_g_ is at ca. 54 °C independently of the polymer concentration. Moreover, the cold crystallization of the PLA occurs at 76 °C with a ΔH_c_ of around 21 J/g in the three formulations, as observed by the exothermic event in Figure 5B. Furthermore, the T_m_ of the NP-PLAs increases from 165 °C to 170 °C when the polymer concentration increases from 2.5 g/L to 7.5 g/L which might be ascribed to the higher proportion of PLA within the sample that enables the formation of bigger crystals during the cold crystallization.

The PLA concentration in the organic phase also influenced the R_H_ of the NPs, although its effect was less pronounced compared to the impact of PLA molecular weight. The formulation with the lowest PLA concentration resulted in the smallest R_H_. Consequently, PLA-NPs obtained with a concentration of 2.5 g PLA/L were chosen for further optimization of the PVA concentration parameter

#### 3.2.3. Effect of PVA Concentration

The effect of PVA concentration was evaluated in NPs formulated using PLA32 and a polymer concentration of 2.5 g/L (Table 7). The R_H_ of the NPs decreased from ca.149 nm to 107 nm as the PVA concentration increased from 1% to 3%, which is related to an increase of the specific surface due to the increase of the surfactant amount. PDI and Z-potential values were around 0.05 and −20, respectively, and were unaffected by the PVA concentration variation.

The 3% PVA formulation was selected for further analysis due to the smaller R_H_ obtained for the formulations. However, given the polymeric nature of PVA, additional investigations were conducted to better understand its effect on the physicochemical properties of the resulting NPs. DSC analysis was performed from room temperature to 230 °C to examine the thermal transitions of the formulated NPs (Figure 5C, Table 8). The T_g_ of NPs was approximately 54 °C and showed minimal variation (less than 2 °C) with increasing PVA concentration, from 1% to 3%. However, higher PVA concentrations induced cold crystallization before the PLA T_g_, likely due to PVA, as evidenced by an increase in ΔH_c_ from 0 to 6 J/g as PVA concentration increased from 1% to 3%. Additionally, the increase in PVA concentration led to a new T_g_ at 31 °C, which can be attributed to the more external regions of the NPs, where PVA is not blended with PLA. This T_g_ is slightly lower than that of pure PVA (47 °C), enabling PVA crystallization. The T_c_ around 76 °C, associated with PLA, remained constant regardless of PVA concentration. However, a decrease in the ΔH_c_ from 21 to 8 J/g was observed, likely due to a reduction in the PLA/PVA ratio within the NPs and the formation of a defective PLA crystalline phase caused by PVA presence.

The T_m_ of both PLA and PVA decreased by 5 °C and 10 °C, respectively, as PVA concentration increased, while the ΔH_m_ of PLA and PVA showed corresponding decreases and increases. These changes suggest that higher PVA concentrations hinder the crystallization of both PVA and PLA. Furthermore, the endothermic signal at 161 °C in the 3% PVA sample displayed a relative minimum at 150 °C (see black arrow in Figure 5C), confirming that increased PVA concentration impedes PLA crystallization in the α-phase.

**Figure 6:**
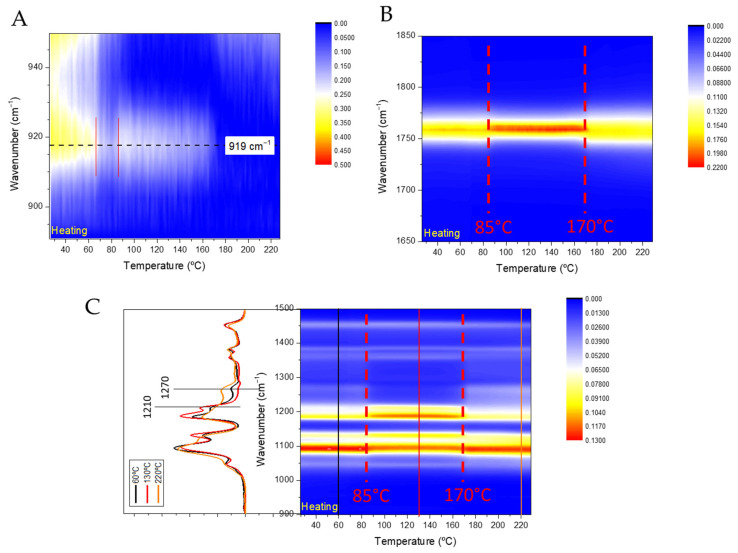
Time-resolved FTIR spectra of the NP-PLA containing 2% PVA. (**A**) 890–950 cm^−1^ region, (**B**) 1650–1850 cm^−1^ region and (**C**) 900–1500 cm^−1^ region.

In addition, the physicochemical state of the NPs was also evaluated by time-resolved FTIR analysis, and the evolution of the polymeric chains was evaluated upon heating. At room temperature, NPs containing 1%, 2% and 3% of PVA had partially crystallized as evidenced by the FTIR broadband at ca. 919 cm^−1^ (Figure 6A, Figures S8A and S9A of the Supporting Information), which was slightly shifted to higher wavenumbers, indicating the presence of mesophase [39]. The intensity of the 919 cm^−1^ band decreased between 65 °C and 85 °C during the PLA cold crystallization (see red lines in Figure 6A, Figures S8A and S9A), and appeared again at 85 °C as a sharper signal centered at 919 cm^−1^, indicating the reorganization of the chains contained in the mesophase to crystallize in the α-phase. Moreover, this band disappeared in the molten state at 160 °C in agreement with the DSC results previously obtained. Moreover, the carbonyl signal at 1750 cm^−1^ shifted to a higher wavenumber during the cold crystallization and shifted back to a lower wavenumber during the PLA melt and presented a shark peak during the crystallization and a Gaussian peak before crystallization and during the melt (see red lines in Figure 6B, Figures S8B and S9B).

Furthermore, in PLA the interchain interaction signal appears at 1210 cm^−1^, and the amorphous phase at 1270 cm^−1^ [39]. The cold crystallization observed by DSC assigned to PLA was confirmed by the decrease at 85 °C of the amorphous phase band at 1270 cm^−1^ and the increase of the interchain interaction band at 1210 cm^−1^, independently of the PVA concentration (Figure 6C, Figures S8C and S9C). Moreover, the intensity of the amorphous phase signal at 1270 cm^−1^ increased upon heating up at 175 °C, 170 °C and 165 °C in the formulations containing 1, 2 and 3% of PVA, respectively, in agreement with the decrease of the PLA T_m_ observed by DSC. The melting of PLA was further indicated by a decrease in the interchain interaction signal at 1210 cm^−1^. Additionally, the transition of the C-H stretching signals at 3000 cm^−1^ was observed, shifting from three distinct peaks to a sharp, singular signal. The melting of PVA was confirmed by an increase in the intensity of the OH stretching signal at 3300 cm^−1^.

### 3.3. Formulation of Loaded-JQ1 NPs: Loading Efficiency, Encapsulation Efficiency and Release Profile

The impact of PVA concentration in NPs optimization on encapsulation efficiency (EE%), loading efficiency (LE%), and the release profile of JQ1 was evaluated. First, optimized formulations using PLA32 and a polymer concentration of 2.5 g/L were employed for JQ1 encapsulation. The characterization of the JQ1-loaded NPs (JQ1-NPs) is presented in Table 9. EE% and LE% were obtained by HPLC after lyophilization and destruction of the loaded NPs.

The increase of PVA percentage in the formulation decreased the EE% from 56% to 41% and the LE from 3,3% to 1,8%, which might be ascribed to the hydrophobic or hydrophilic character of the formulation components. PLA is a hydrophobic polymer, whilst PVA is a hydrophilic polymer. Moreover, JQ1 is preferentially hydrophobic, and therefore the increase of the PVA concentration disrupts the proper interaction between PLA and JQ1, leading to a reduction in EE%.

**Figure 7:**
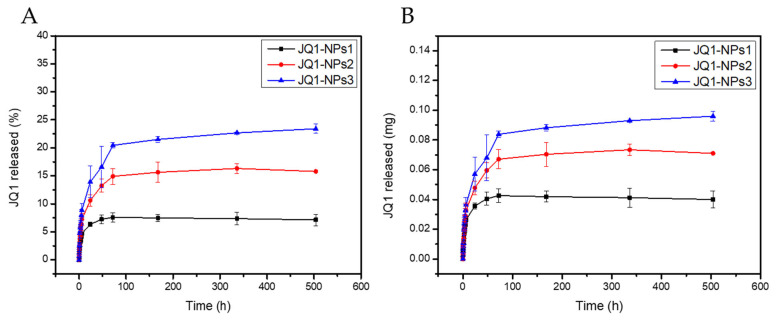
Relative (**A**) and absolute (**B**) quantity of JQ1 released from nanoparticles containing different percentages of PVA.

Finally, in vitro release studies from the three formulations containing 1, 2, and 3% PVA were conducted. The release profiles were determined using the dialysis bag method at pH 7.4 and 37 °C to mimic physiological conditions [16]. Figure 7 shows the expected triphasic release profile for any formulation [26]. Results indicate that the burst release was negligible, and the release over time was very controlled, which can be attributed to the high stability of the NPs. Notably, the JQ1-NPs3 released nearly 10% of JQ1 within the first 5 h, while the JQ1-NPs1 released less than 5% of JQ1 (Figure 7A). The variation in the percentage of JQ1 released may be related to the decrease in EE% as PVA concentration increases. However, the absolute release of JQ1 (in mg) was faster with higher PVA concentrations (Figure 7B). These results suggest that the hydrophilic nature of PVA enhances the water swelling of the NPs, thereby increasing JQ1 release. This indicates that the amount of JQ1 released can be tailored by adjusting the PVA concentration during formulation.

## 4. Discussion

BET proteins were discovered in the early 1990s [3,43] and in the last decade BET inhibitors have emerged as a novel strategy to treat TNBC [25]. Since then, several compounds have been developed and are currently under clinical development [44]. Among BET inhibitors, JQ1 has demonstrated strong anti-cancer activity in preclinical models in hematological and solid tumors [44,45]. However, JQ1 presents a relatively short half-life in vivo (approximately 1 h in CD1 mice) due to a rapid clearance by cytochrome P450 enzymes that limits its effectiveness in long-term treatments requiring frequent dosing to maintain therapeutic levels. Moreover, JQ1 present a high LogP (6.41) value indicating a significant lipophilicity which might limit its solubility and distribution. In addition, JQ1 inhibits the BET family proteins in a non-selective manner which can result in off-target effects and resistance development [45,46]. Addressing these disadvantages requires a multi-disciplinary approach, including pharmacology, molecular biology, and material science. Different strategies have been proposed to improve the efficacy of JQ1 such as combination therapies [27,47], identifying biomarkers to predict selectivity to JQ1 [4] and optimising pharmacokinetics [26]. Optimizing the pharmacokinetics using polymeric NPs is a promising strategy that enables drug-controlled release of drugs and targeted delivery, improving efficacy while reducing side effects [7]. Particularly, the use of FDA-approved polymers such as PLGA or PLA, is a well-established strategy to optimize DDS since, besides their biocompatibility and biodegradability properties, they assure prompt translation to the clinics [8]. PLA is a more hydrophobic polymer than PLGA due to the lack of glycolic acid units and, therefore, is suitable for encapsulating hydrophobic drugs such as JQ1. Moreover, the hydrophobic character of PLA extends its degradation under physiological conditions and is preferable when a more sustained release is required. These features position PLA as an ideal candidate for the development of advanced nanocarrier systems for the encapsulation of JQ1 [9]. However, contradictory results are often found in literature due to a lack of understanding of the formulation parameters effect on the final release profile. Moreover, the synthesis of PLA at the industrial level is usually accomplished using Sn-derived catalysts, which is potentially harmful due to its accumulation in brain and lung tissue, limiting its application in the biomedical field. Therefore, the synthesis of well-controlled PLA macrostructures using biocompatible catalytic systems, which enables good control over the polymerization process is required.

The main objective of this study was to optimize formulations for converting JQ1 into nanomedicines. This was achieved by evaluating the impact of various parameters on the R_H_, PdI, and Z-potential of the formulations. Additionally, the physicochemical state of the NPs was assessed, as it plays a critical role in determining the release profile of JQ1.

In the first part of the article PLA derivatives presenting different molecular weights were synthesized through a Zn-based initiator presenting high monomer conversion and good control of the molecular weight (Figure 1 and Table 1). The organometallic compound containing Zn as metal was selected attending the biocompatibility characteristics of Zn, since traces of the catalyst might be trapped within the polymer structure. Then, the influence of the molecular weight on the physicochemical properties of the polymers was evaluated by TGA and DSC (Figure 2). The molecular weight significantly affected the degradation temperature, likely due to the entrapment of the catalyst and unreacted monomers within the polymer matrix. In addition, the T_g_ temperature remained mainly constant with the increase of the molecular weight. However, the increase of the PLA molecular weight affected to physicochemical state of the polymer as evidenced by the double melting peak observed by DSC, suggesting that the crystallization of PLA is hampered by the increase of the molecular weight. The double melting peak observed could be ascribed to the partial crystallization of the polymer in a defective crystal phase, which may affect the release rate (Table 2).

Subsequently, polymeric NPs were formulated by a double emulsion method (Figure 3), obtaining an R_H_ of ca. 150 nm and monodispersed distributions (Table 3), which remained constant for 4 weeks stored at 4 °C (Figure 4). Three parameters of the formulation process were analyzed to understand their effect on the final physicochemical state: the PLA molecular weight (PLA-Mw), PLA concentration in the organic phase, and finally the PVA concentration in the aqueous phase.

The variation in PLA-Mw had a significant impact on the size of the NPs, with lower PLA-Mw proving to be the most suitable raw material for formulation. This is because it resulted in smaller and more homogeneously dispersed nanodevices (Table 3). DSC analysis of the different polymeric NPs (Figure 5A) showed negligible differences compared to the raw PLA (Table 4), regardless of molecular weight, which follows previous reports [48]. The effect of PLA concentration on the formulation process was less pronounced. However, the lower PLA concentration (2.5 g/L) proved optimal for fine-tuning the polymeric formulations (Table 5). The thermodynamic state of the NPs after formulation was found to be independent of the polymer concentration used, as evidenced by DSC analysis (Table 6 and Figure 5B). On the other hand, the PVA concentration had a definitive impact on the formulation process. While PDI and Z-potential values remained unaffected by variations in PVA concentration, the size of the NPs decreased with increasing PVA concentration (Table 7), which is attributed to the high surface/volume ratio obtained as the PVA concentration increases. The effect of PVA concentration was further analyzed through the thermodynamic state of the NPs (Table 8 and Figure 5C), which suggested that higher PVA concentrations hamper the crystallization of PLA, forcing the formation of the defective α’-phase (Figure 5C).

Once the formulation process was optimized, we proceeded with the encapsulation of the BETi inhibitor JQ1. The main challenge was to tailor the release of the drug and loading by fine-tuning three key variables identified as critical for encapsulation: PLA-Mw, PLA concentration, and PVA concentration. Based on our results, we determined that a lower PLA Mw, combined with a polymer concentration of 2.5 g/L, was optimal for further encapsulation processes. However, the PVA concentration appeared to be a decisive factor for drug loading efficiency and release. Therefore, various PVA concentrations were systematically studied, 1–3% PVA. Due to the high hydrophobic character of JQ1, a PVA concentration of 1% resulted in higher EE% and LE%. The results suggest that higher PVA concentrations disrupt interactions between the PLA core and JQ1, potentially reducing both EE% and LE% (Table 9).

Finally, a triphasic release profile of JQ1 was observed for the three PVA-dependent formulations, indicating successful optimization (Figure 7). The negligible burst release further supports the stability of the formulations. Additionally, the results demonstrated that the release rate of the drug could be modulated by varying the PVA concentration. Higher PVA concentrations led to faster drug release, likely due to the hydrophilic nature of PVA, which enhances water diffusion into the nanoparticle core.

## 5. Conclusions

This study highlights the potential of PLA-derived NPs as advanced DDS for the BET inhibitor JQ1. By synthesizing PLA derivatives using a biocompatible Zn-based catalyst, we achieved high monomer conversion and controlled molecular weight. Optimizing the formulation process through a systematic evaluation of PLA-Mw, PLA concentration, and PVA concentration demonstrated that lower PLA-Mw and a polymer concentration of 2.5 g/L generated NPs with adequate size and stability. PVA concentration emerged as a critical parameter, with a concentration of 1% yielding the highest EE% and LE%, while higher concentrations disrupted interactions between JQ1 and the PLA core. The complexity of the formulation process requires the evaluation of several parameters that might also influence the release profile of JQ1 and that will be addressed in future perspectives, although the present study is focused on three to maintain the clarity of the conclusions.

Overall, the findings underscore the importance of understanding and optimizing formulation parameters to develop efficient and scalable nanoparticle-based delivery systems for hydrophobic drugs like JQ1. This work pays the way for future clinical translation by using FDA-approved polymers and biocompatible catalytic systems, ensuring safety and effectiveness in biomedical applications.

## Figures and Tables

**Table 1:** Synthesis of PLA derivatives.

Sample Name	[LA]/[Init.]	Conversion (%)	Mw_(theo.)_ (Da) ^a^	Mw_(exp.)_ (Da) ^b^	Mn_(exp.)_ (Da) ^b^	PDI ^b^
PLA32	210	80	30,000	32,823	16,985	1.932
PLA56	390	87	55,000	56,996	27,992	2.035
PLA71	490	85	70,000	71,286	40,714	1.751
PLA98	660	84	95,000	98,463	67,112	1.467

Polymerization conditions: 25 mol of Initiator, toluene as a solvent at 90 C. ^a^ Theoretical Mn = (monomer/Initiator) × (% conversion) × (Mw of LA). ^b^ Determined by GPC relative to polystyrene standards in chloroform.

**Table 2:** Thermal transitions (T_g_, T_c_ and T_m_) and enthalpies (ΔHc, ΔHm) of the different PLA obtained.

Polymer	Mw (kg/mol)	T_d_ (°C)	T_g_ (°C)	T_cc_ (°C)	ΔH_c_ (J/g)	T_m_^1^ (°C)	T_m_^2^ (°C)	ΔH_m_ (J/g)
PLA32	33	282.1	68.01	-	-		172.15	62.741
PLA56	56	363.5	68.55	-	-		175.77	54.494
PLA71	71	390.3	73.85	-	-	159.57	173.04	68.225
PLA98	98	322.1	70.84	-	-	159.80	174.81	67.935
PVA	31–55	-	47.06	-	-	210.17	-	50.222

T_g_ (Glass Transition Temperature); T_c_ (Crystallization Temperature); T_m_ (Melting Temperature); ΔH_c_ (Crystallization Enthalpy); ΔH_m_ (Melting Enthalpy). The numbers indicate that those transitions are the first or the second one from lower to higher temperatures.

**Table 3:** Characterization of PLA-NPs obtained by different molecular weight PLAs as raw material.

Polymer	R_H_ (nm)	PdI	Z Potential (mV)
PLA32	149.5 ± 1.2	0.07 ± 0.01	−18.1 ± 0.513
PLA56	168.5 ± 8.1	0.19 ± 0.01	−19.6 ± 0.379
PLA71	180.1 ± 6.5	0.15 ± 0.02	−21.9 ± 0.252
PLA98	176.4 ± 2.7	0.14 ± 0.03	−17.8 ± 0.451

R_H_ (Hydrodinamic radio); PdI (Polydispersity index).

**Table 4:** Thermal transitions (T_g_, T_cc_ and T_m_) and enthalpies (ΔH_c_, ΔH_m_) of the different PLA-NPs obtained by the different molecular weight PLAs.

Polymer	[PLA] (g/L)	[PVA] (%)	T_g_ (°C)	T_cc_ (°C)	ΔH_c_ (J/g)	T_m_ (°C)	ΔH_m_ (J/g)
PLA32	2.5	1	53.71	76.22	21.54	165.99	41.46
PLA56	2.5	1	58.33	81.56	20.28	173.54	37.14
PLA71	2.5	1	58.51	83.31	16.506	173.34	35.50
PLA98	2.5	1	59.58	83.31	18.39	174.49	39.56

T_g_ (Glass Transition Temperature); T_c_ (Crystallization Temperature); T_m_ (Melting Temperature); ΔH_c_ (Crystallization Enthalpy); ΔH_m_ (Melting Enthalpy).

**Table 5:** Characterization of PLA32-NPs as a function of polymer concentration.

Polymer Concentration (g/L)	R_H_ (nm)	PdI	Z Potential (mV)
2.5	149.5 ± 1.2	0.07 ± 0.01	−18.1 ± 0.513
5	153.4 ± 2.554	0.091 ± 0.013	−17.3 ± 0.173
7.5	168.2 ± 2.098	0.091 ± 0.014	−14.5 ± 0.361

R_H_ (Hydrodinamic radio); PdI (Polydispersity index).

**Table 6:** Thermal transitions (T_g_, T_cc_ and T_m_) and enthalpies (ΔHc, ΔHm) of the different PLA32-NPs obtained at different PLA concentrations.

[PLA] (g/L)	[PVA] (%)	T_g_ (°C)	T_cc_ (°C)	ΔH_c_ (J/g)	T_m_^PLA^ (°C)	T_m_^PVA^ (°C)	ΔH_m_^PLA^ (J/g)	ΔH_m_^PVA^ (J/g)
2.5	1	53.71	76.22	21.54	165.99	213.29	41.46	7.98
5	1	53.35	76.77	20.76	166.89	213.98	41.08	9.11
7.5	1	54.62	76.69	22.91	170.22	204.25	44.53	4.65

T_g_ (Glass Transition Temperature); T_c_ (Crystallization Temperature); T_m_ (Melting Temperature); ΔH_c_ (Crystallization Enthalpy); ΔH_m_ (Melting Enthalpy).

**Table 7:** Characterization of PLA32-NPs as a function of PVA concentration.

PVA Concentration	R_H_ (nm)	PdI	Z Potential (mV)
1%	149.5 ± 1.2	0.07 ± 0.01	−18.1 ± 0.513
2%	114.7 ± 2.90	0.04 ± 0.02	−23.0 ± 0.755
3%	107.2 ± 0.70	0.07 ± 0.03	−17.0 ± 0.808

R_H_ (Hydrodinamic radio); PdI (Polydispersity index).

**Table 8:** Thermal transitions (T_g_, T_c_ and T_m_) and enthalpies (ΔHc, ΔHm) of the different PLA32-NPs obtained at different ratios of PVA.

[PVA] (%)	T_g_^1^ (°C)	T_cc_^1^ (°C)	ΔH_cc_^1^ (J/g)	T_g_ (°C)	T_cc_ (°C)	ΔH_cc_ (J/g)	T_m_^PLA^ (°C)	T_m_^PVA^ (°C)	ΔH_m_^PLA^ (J/g)	ΔH_m_^PVA^ (J/g)
1	-	-	-	53.71	76.22	21.54	165.99	213.29	41.46	7.98
2	-	45.92	2.94	54.26	75.65	14.27	163.41	207.51	32.93	16.24
3	31.07	45.75	5.78	55.10	76.50	8.01	161.97	203.86	20.31	21.606

T_g_ (Glass Transition Temperature); T_c_ (Crystallization Temperature); T_m_ (Melting Temperature); ΔH_c_ (Crystallization Enthalpy); ΔH_m_ (Melting Enthalpy). The numbers indicate that those transitions are the first or the second one from lower to higher temperatures.

**Table 9:** Characterization of JQ1-loaded NPs as a function of PVA concentration.

Formulation	PVA (%)	R_H_ (nm)	PdI	Z Potential (mV)	EE%	LE%
JQ1-NPs1	1	176 ± 3694	0.016 ± 0.01	−16.5 ± 0.503	55.84 ± 0.05	3.36 ± 0.004
JQ1-NPs2	2	133 ± 2203	0.088 ± 0.018	−18.0 ± 1.01	44.60 ± 0.03	2.59 ± 0.001
JQ1-NPs3	3	1222 ± 1587	0.017 ± 0.092	−16.7 ± 0.153	41.26 ± 0.02	1.88 ± 0.001

R_H_ (Hydrodynamic radio); PdI (Polydispersity index); EE% (encapsulation efficiency); LE% (loading efficiency).

## Data Availability

Data is available within the article.

## Supplementary Materials

The following supporting information can be downloaded at: https://www.mdpi.com/article/10.3390/polym17010123/s1, Figure S1. ^1^H NMR spectrum (500 MHz, 298 K, CDCl_3_) of PLA32 after precipitation; Figure S2. ^1^H NMR spectrum (500 MHz, 298 K, CDCl_3_) of PLA32; Figure S3. GPC analysis of PLA32; Figure S4. GPC analysis of PLA56; Figure S5.GPC analysis of PLA71; Figure S6. GPC analysis of PLA98; Table S1. Main FTIR vibration bands associated with both PLA and PVA; Figure S7. dTGA thermogram of the synthesized PLA-derivatives at a heating rate of 10 °C/min; Figure S8. Time-resolved FTIR spectra of the NP-PLA containing 1% PVA; Figure S9. Time-resolved FTIR spectra of the NP-PLA containing 3% PVA.
