# Supplementary material for: Fine-Tuning the Physicochemical Properties of Poly(lactic Acid) Nanoparticles for the Controlled Release of the BET Inhibitor JQ1: Influence of PVA Concentration
Source: Polymers (Basel). 2025 Jan 6;17(1):123. doi: 10.3390/polym17010123 (PMC11722895; doi:10.3390/polym17010123)
Supplement: Supplementary file 1 [file polymers-17-00123-s001.zip › polymers-3412483-supplementary.pdf]

Supplementary Information

Fine-Tuning the Physicochemical Properties of Poly(lactic Acid) Nanoparticles for the Controlled Release of the BET Inhibitor JQ1: Influence of PVA Concentration

Nedjla Kedjar [1,†], Eleonora Iannuzzi [2,†], Martin Kreuzer [3], Carlos Alonso-Moreno [2,*] and Carmen Moya-Lopez [2,*]

1 Laboratory of Applied Chemistry (LAC), Faculty of Sciences Technology, University of Ain Temouchent Belhadj Bouchaib, Ain Temouchent 46000, Algeria; njkedj@gmail.com
2 Facultad de Farmacia-Centro de Innovación en Química Avanzada (ORFEO-CINQA), Unidad nanoDrug, Departamento de Química Inorgánica, Orgánica y Bioquímica, Universidad de Castilla-La Mancha, 02071 Albacete, Albacete, Spain; eleonora.iannuzzi@uclm.es
3 ALBA Synchrotron, Carrer de la Llum 2-26, 08290 Cerdanyola del Vallès, Barcelona, Spain; mkreuzer@cells.es
* Correspondence: carlos.amoreno@uclm.es (C.A.-M.); camen.moya@uclm.es (C.M.-L.); Tel.: +34-92-629-5300 (ext. 3499) (C.A.-M.); +34-92-629-5300 (ext. 3499) (C.M.-L.)
† These authors contributed equally to this work.

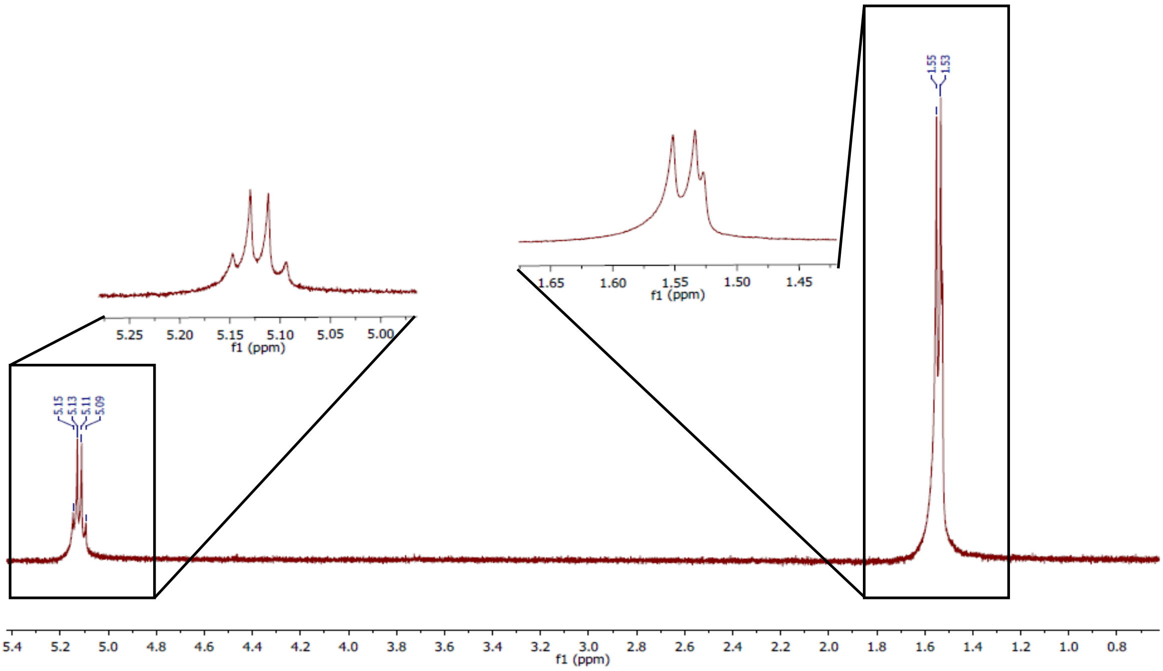

Figure S1. ^{1}H NMR spectrum (500 MHz, 298 K, $CDCl_3$) of PLA32 after precipitation.

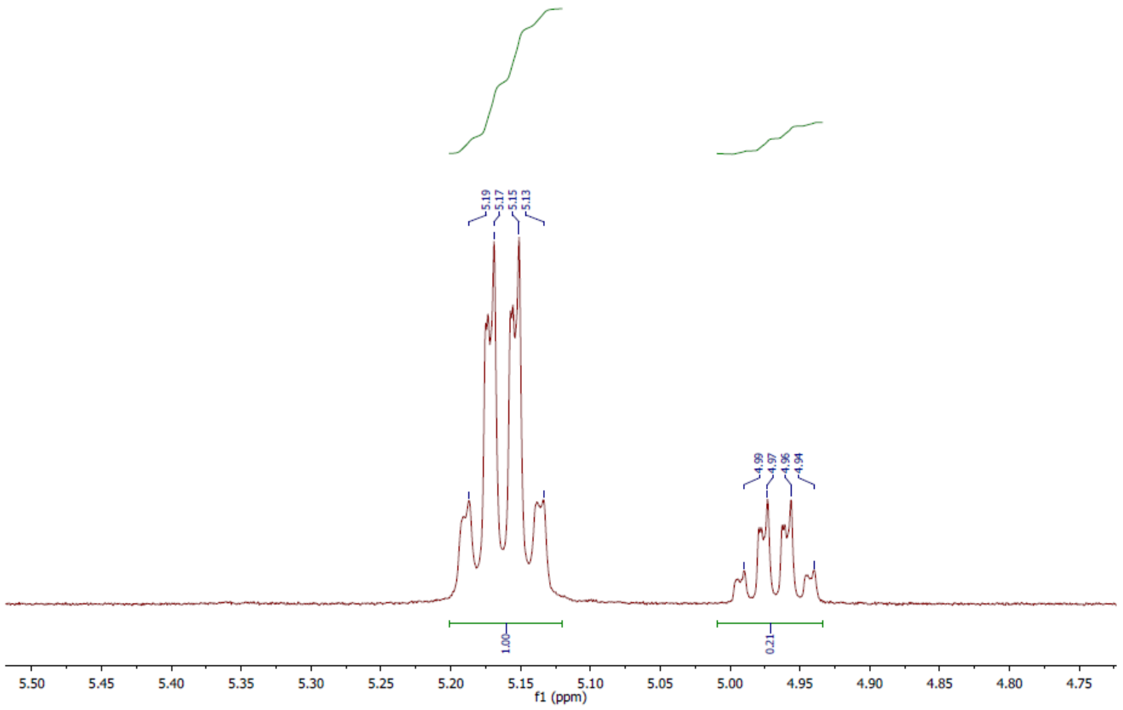

Figure S2. ^{1}H NMR spectrum (500 MHz, 298 K, $CDCl_3$) of PLA32

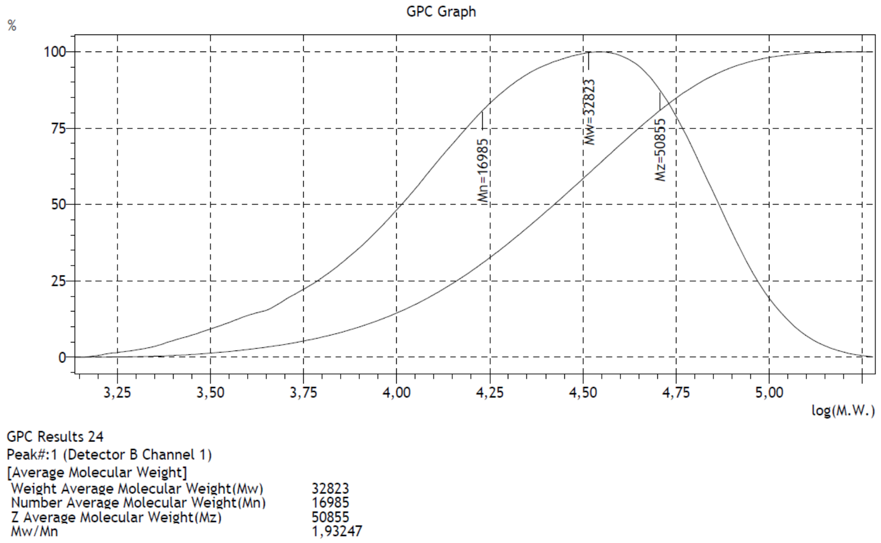

Figure S3. GPC analysis of PLA32

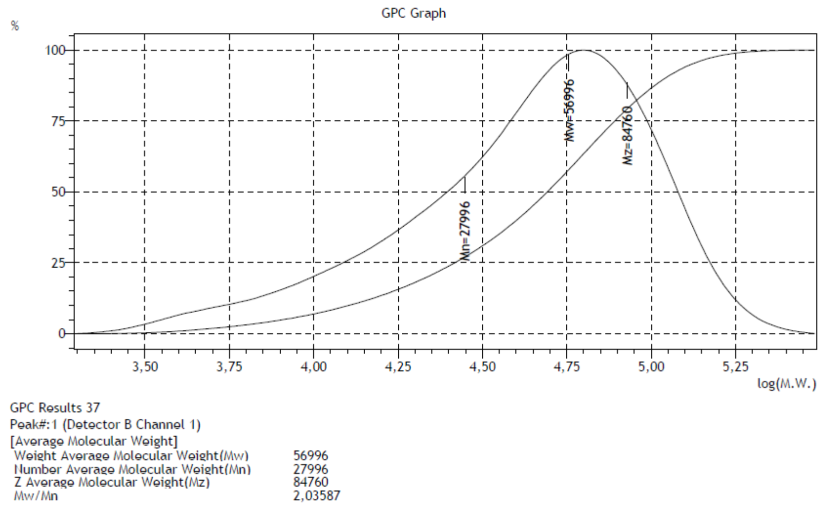

Figure S4. GPC analysis of PLA56

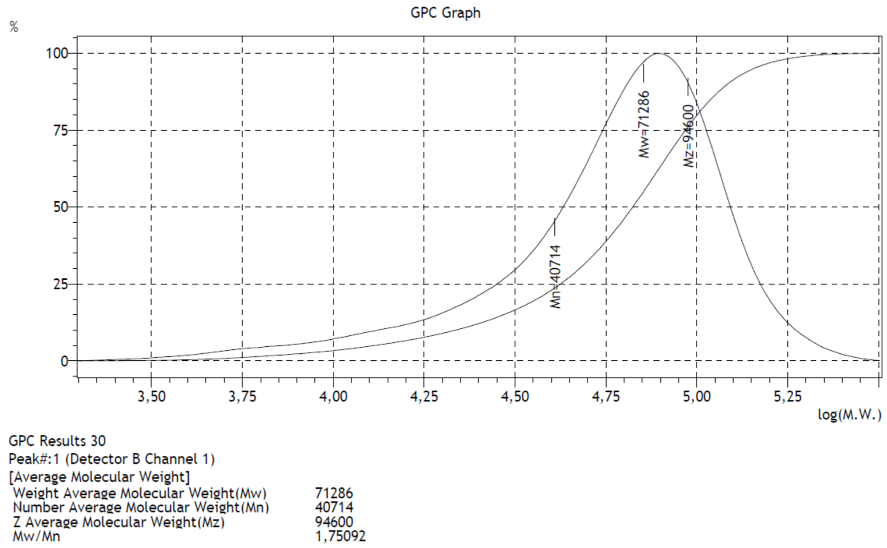

Figure S5.GPC analysis of PLA71

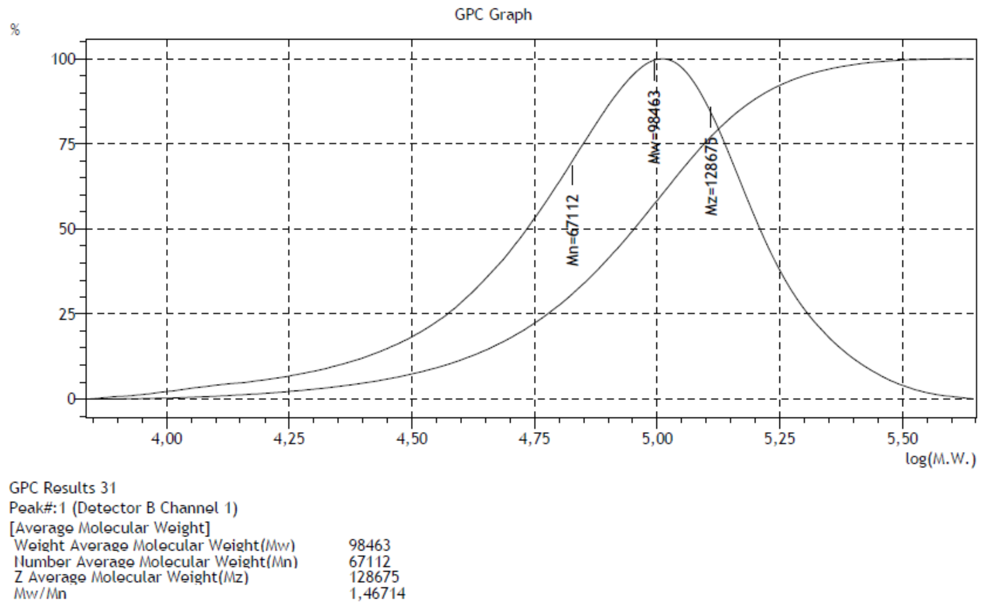

Figure S6. GPC analysis of PLA98

Table S1. Main FTIR vibration bands associated with both PLA and PVA.

PLA		PVA	
Assignements	Wavenumber	Assignements	Wavenumber
ν_{as} CH_3	2993	OH	3300
ν C=O	1750	C-O	1030
δ_{as} CH_3	1454		

δ_s CH_3	1384		
δ_1 CH_3 + d_sCH_3	1358		
δ CH +ν COC	1270		
ν_{as} COC	1210		
γ_{as} CH_3	1129		
ν_s COC	1083		
ν C-CH_3	1043		
10_3 helix	920		

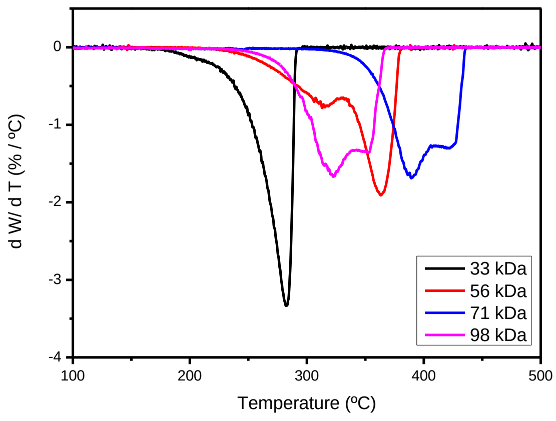

Figure S7. dTGA thermogram of the synthesized PLA-derivatives at a heating rate of 10°C/min

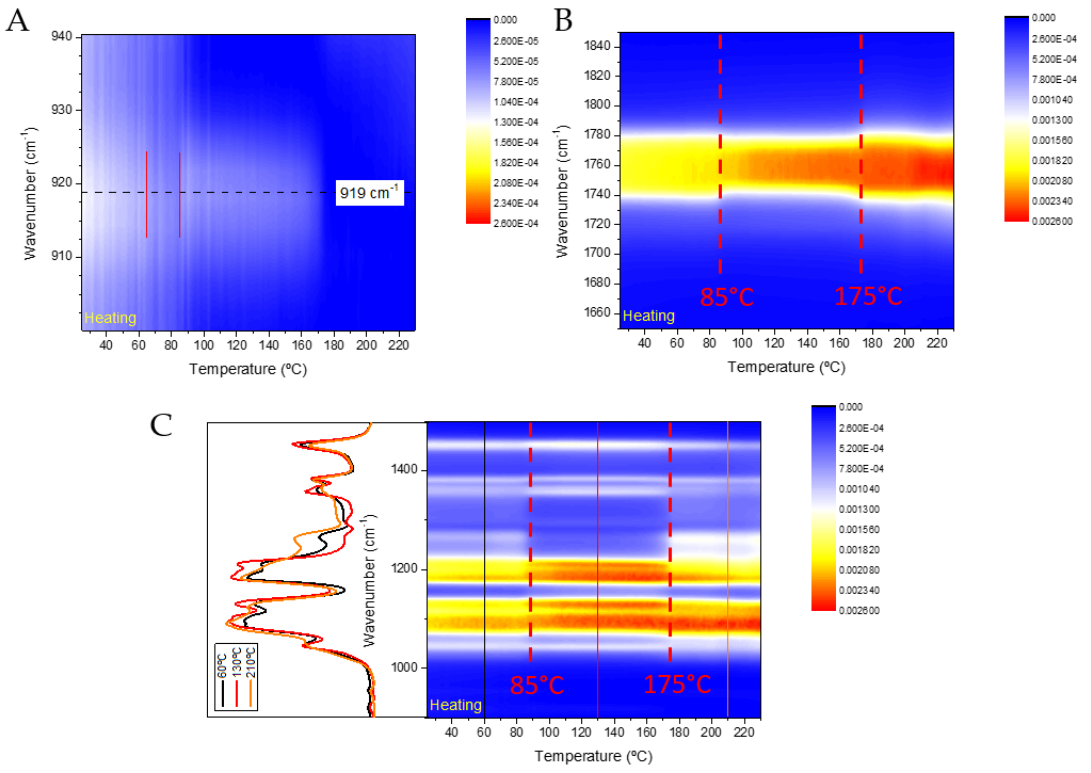

Figure S8. Time-resolved FTIR spectra of the NP-PLA containing 1% PVA

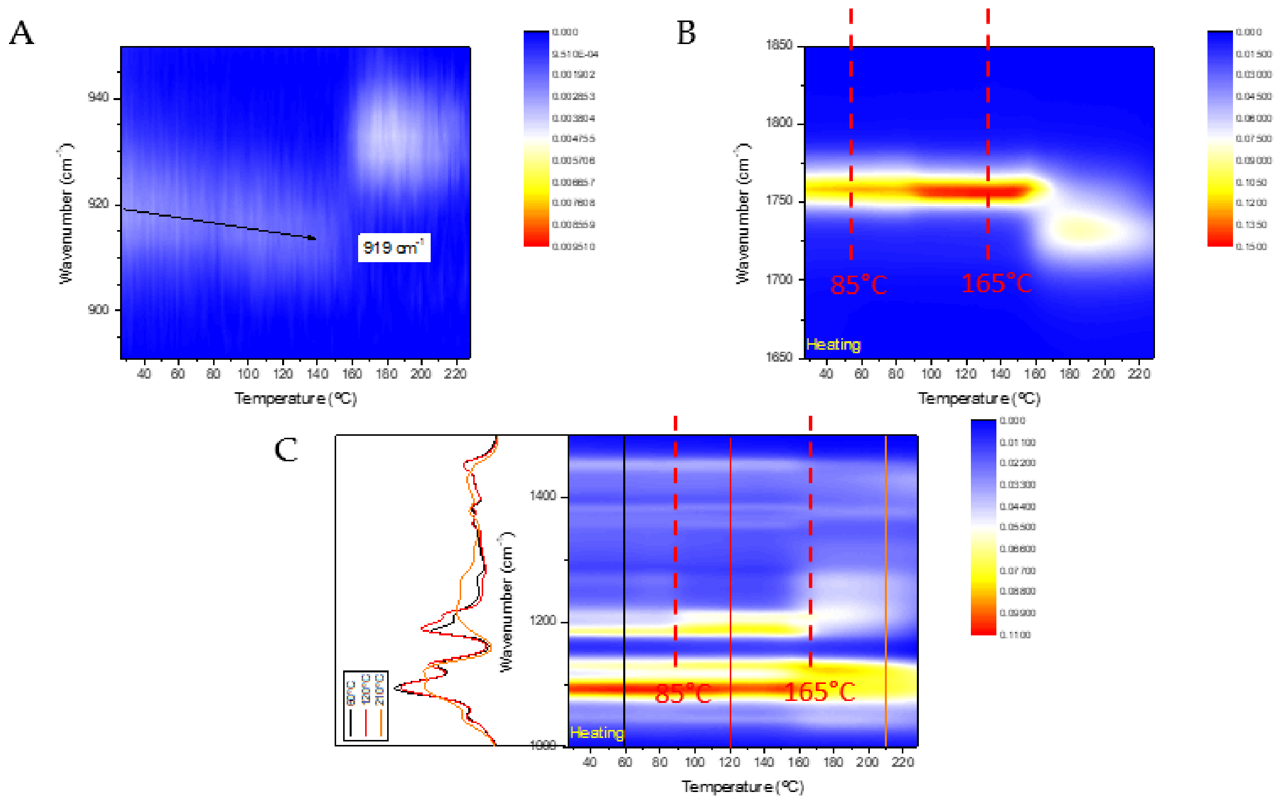

Figure S9. Time-resolved FTIR spectra of the NP-PLA containing 3% PVA
